# Gridlock from diagnosis to treatment of multidrug-resistant tuberculosis in Tanzania: low accessibility of molecular diagnostic services and lack of healthcare worker empowerment in 28 districts of 5 high burden TB regions with mixed methods evaluation

**DOI:** 10.1186/s12889-019-6720-6

**Published:** 2019-04-11

**Authors:** Stellah G. Mpagama, Peter M. Mbelele, Anna M. Chongolo, Isaack A. Lekule, Johnson J. Lyimo, Gibson S. Kibiki, Scott K. Heysell

**Affiliations:** 1 Kibong’oto Infectious Diseases Hospital, Mae Street, Lomakaa road, Sanya Juu, Siha Kilimanjaro, Tanzania; 2 Kilimanjaro Clinical Research Institute/Kilimanjaro Christian Medical University College, Moshi, Tanzania; 3 Nelson Mandela African Institution of Science and Technology, Arusha, Tanzania; 4 National TB and Leprosy Programme, Dar es Salaam, Tanzania; 5 East African Health Research Commission, Bujumbura, Burundi; 6 Division of Infectious Diseases and International Health, University of Virginia, Charlottesville, USA

**Keywords:** Multidrug resistant tuberculosis (MDR-TB), Human immunodeficiency virus (HIV), Molecular diagnostics, Health Systems, Health care workers, Implementation barriers

## Abstract

**Background:**

Multidrug-resistant tuberculosis (MDR-TB) outcomes are adversely impacted by delay in diagnosis and treatment.

**Methods:**

Mixed qualitative and quantitative approaches were utilized to identify healthcare system related barriers to implementation of molecular diagnostics for MDR-TB. Randomly sampled districts from the 5 highest TB burden regions were enrolled during the 4th quarter of 2016. District TB & Leprosy Coordinators (DTLCs), and District AIDS Coordinators (DACs) were interviewed, along with staff from all laboratories within the selected districts where molecular diagnostics tests for MDR-TB were performed. Furthermore, the 2015 registers were audited for all drug-susceptible but retreatment TB cases and TB collaborative practices in HIV clinics, as these patients were in principal targeted for drug susceptibility testing by rapid molecular diagnostics.

**Results:**

Twenty-eight TB districts from the 5 regions had 399 patients reviewed for retreatment with a drug-susceptible regimen. Only 160 (40%) had specimens collected for drug-susceptibility testing, and of those specimens only 120 (75%) had results communicated back to the clinic. MDR-TB was diagnosed in 16 (13.3%) of the 120 specimens but only 12 total patients were ultimately referred for treatment. Furthermore, among the HIV/AIDS clinics served in 2015, the median number of clients with TB diagnosis was 92 cases [IQR 32–157] yet only 2 people living with HIV were diagnosed with MDR-TB throughout the surveyed districts. Furthermore, the districts generated 53 front-line healthcare workers for interviews. DTLCs with intermediate or no knowledge on the clinical application of XpertMTB/RIF were 3 (11%), and 10 (39%), and DACs with intermediate or no knowledge were 0 (0%) and 2 (8%) respectively (*p* = 0.02). Additionally, 11 (100%) of the laboratories surveyed had only the 4-module XpertMTB/RIF equipment. The median time that XpertMTB/RIF was not functional in the 12 months prior to the investigation was 2 months (IQR 1–4).

**Conclusions:**

Underutilization of molecular diagnostics in high-risk groups was a function of a lack of front-line healthcare workforce empowerment and training, and a lack of equipment access, which likely contributed to the observed delay in MDR-TB diagnosis in Tanzania.

**Electronic supplementary material:**

The online version of this article (10.1186/s12889-019-6720-6) contains supplementary material, which is available to authorized users.

## Background

For the fourth consecutive year, tuberculosis (TB) is the leading killer from an infectious disease worldwide, and despite curative antibiotic treatment, remains the leading cause of death for people living with human immunodeficiency virus (PLWH) [1]. The confluence of HIV and poorly integrated health systems in Tanzania, not unlike other countries in sub Saharan Africa, has fueled a resurgence in TB including multidrug resistant (MDR)-TB [2]. MDR-TB is defined as in-vitro resistance of *Mycobacterium tuberculosis* to at least isoniazid and rifampicin and is associated with worse treatment outcomes, higher mortality and longer treatment durations than drug-susceptible TB [3]. The World Health Organization (WHO) declared MDR-TB as a public health crisis with need to scale up diagnosis of MDR-TB, increase the number of patients initiated on MDR-TB treatment and control the ongoing transmission [4]. Strategies for scale up include universal drug-susceptibility testing and delivery of this laboratory capacity closer to the point of care, with initial focus on those patients at higher risk for MDR-TB such as those co-infected with HIV or with a prior history of TB treatment, so-called “retreatment” cases. While critical to implement, these strategies place a significant burden of agency on the front-line healthcare worker to recognize the need for novel diagnostics, access and utilize novel diagnostics effectively, and support patients to bridge to proper treatment. In many situations, this burden represents an unfunded mandate.

The WHO estimates that for the Africa region only 48% of incident cases of MDR-TB were actually detected in 2014, and among those detected in the same year, only 68% were started treatment [1]. Prior to 2012, the health systems in Tanzania relied on smear microscopy for TB diagnosis, and only in rare cases was MDR-TB established by conventional culture-based phenotypic methods which took up to 3 months for results and which were rarely available for practical clinical care. In 2009, MDR-TB treatment was made available through a national referral process, and molecular diagnostics were simultaneously being commercially distributed and endorsed by the WHO and other stakeholders. Excluding assays in the research setting, current molecular diagnostics available for clinical care in Tanzania are the Xpert MTB/RIF (Cepheid, USA), and the Genotype MTBDR*plus* (Hain Lifescience, Germany). The Genotype MTBDR*plus* screens for both isoniazid *(inhA* and *katG* mutation) and rifampicin (*rpoB* mutation) resistance and requires at least 2 days for completion. The Xpert MTB/RIF assay can be completed in 2 h and requires debatably less technical expertise, but only detects rifampin resistance [5].

Although these molecular diagnostics have desired accuracy and capacity to produce rapid results for clinical action, in other settings outside of Tanzania the effect has neither been transformed into rapid initiation of treatment nor improved patients outcomes [6–8]. Using 2016 Global Burden of Disease data investigators have recently attributed 5.0 million excess deaths in low and middle income countries were attributable to receipt of poor-quality care and 3.6 million were due to non-utilization of existing healthcare services [9]. We hypothesized that a lack of improvement in TB outcomes in Tanzania was similarly symptomatic of poor quality care and non-utilization of existing healthcare, and that understanding implementation barriers faced by front-line healthcare workers may produce remediable solutions [10, 11]. This mixed-methods study was part of a nationwide series of investigations supported by the WHO that we have termed “gridlock from MDR-TB diagnosis to treatment,” to inform strategies for optimal use of molecular diagnostic technology to control the TB epidemic and reshape the healthcare workforce.

## Methods

### Setting

The 5 regions with highest burden of TB in Tanzania; Dar es Salaam, Mwanza, Shinyanga, Mbeya and Morogoro were included. Tanzania is estimated to have a population of approximately 45 million according to the 2012 Population National Census and the studied regions reflected a catchment area of 30% of total population. Due to the dense population and reflective of administrative designations, Dar es Salaam was further sub-divided into 3 designated regions; Ilala, Temeke and Kinondoni [12]. Tanzania’s TB and HIV programmes operate under the preventive directorate-Ministry of Health and are organized into national, regional and district levels. The study involved the districts facilities where most people with TB or TB/HIV are treated. A nationwide survey of 2012 estimated the country prevalence of bacteriologically confirmed TB was 293 (95%CI; 228–358) per 100,000 adult population [13], while the estimated burden of MDR-TB was 1.1 and 3.1% in new and retreatment TB cases respectively [1].

### Study design and participants

**Fig. 1:**
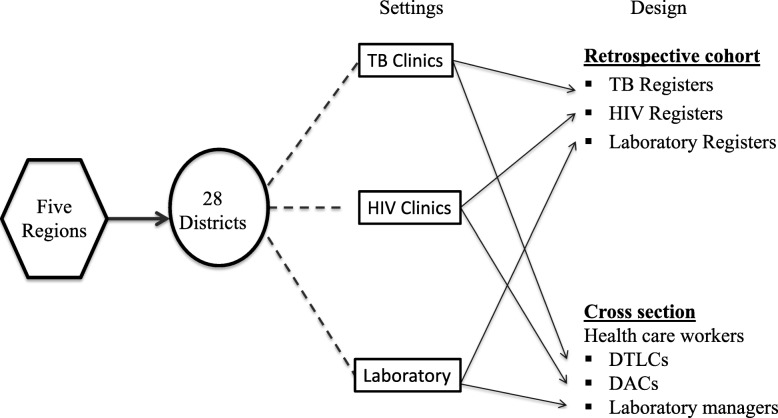
Each region has 5–11 districts. Four districts were randomly selected in each region

Among the purposefully selected high burden TB regions in Tanzania, we instituted a random sampling frame of 55 districts. The numbers of districts in respective regions were as follows: Dar es salaam – Ilala 11 (20% of the total districts), Dar es Salaam – Temeke 8 (15%) and Dar es Salaam-Kinondoni 9 (16%), Mbeya 8 (14%), Morogoro 7 (13%), Mwanza 7 (13%) and Shinyanga 5 (9%). Of those, four districts were randomly selected from each region using the Fisher-Yates shuffle method [14]. The district was the main study unit and we performed a mixed design; quantitative and qualitative approach using a retrospective cohort and cross-sectional designs (Fig. 1) [15]. For the retrospective cohort design, TB patients registered as retreatment cases and PLWH that attended those randomly selected districts in the previous year, 2015, were audited. While for the cross-sectional component the selected participants were the district coordinators for TB and leprosy (DTLCs) responsible for the district TB clinics or district AIDS coordinators (DACs) responsible for district HIV clinics, and the laboratory managers or designated head of the laboratories performing molecular diagnostics for MDR-TB diagnosis. A component of qualitative approach was used to collect information describing the reasons for the observed findings.

### Study procedure, data management and quality control

We formed a team of Tanzanian clinicians with extensive knowledge and at least 3 years experience working in TB/HIV and MDR-TB clinical management. The team received 3 days of protocol training prior to performing surveys onsite in the districts. Additionally, a pilot of data collection tools was conducted in the districts of a non-selected region, Kilimanjaro, prior to the training of the team. Four types of data collection tools were developed including three unique questionnaires for DTLCs, DACs, or Laboratory Managers, and an extraction tool for information retrieved from the programme records of patients with registered with retreatment TB or PLWH. Various programmatic records were searched such as registers for drug resistant (DR)-TB, the district TB clinic charts, and molecular diagnostics reports from the laboratory and clinics. DTLCs and DACs had common interviews regarding general knowledge of the TB molecular diagnostics including a description of the technology, clinical application and interpretation of the test results. DACs had additional questions on integrated TB/HIV services including those for MDR-TB in PLWH. The laboratories assessment focused on the capacity of equipment for MDR-TB diagnosis and associated challenges that may contribute in reliability of MDR-TB services in the population. Opinions on the challenges or bottlenecks related to MDR-TB diagnosis to treatment were elucidated from all healthcare workers.

The participating healthcare workers’ informed consent process was waived however strict adherence to coding and de-identification of study documents preserved participants confidentiality. The protocol was approved by the WHO - Ethical Review Committee (protocol ID, B40121) and the local Institutional Health Research Ethics Committee (Authors Institute).

### Statistical methods

We estimated the sample size by adopting the recommendation from Lemeshow sampling techniques for evaluating health parameters in developing countries [16]. At least 20 clusters or districts were required for reliable and accurate results. Data analysis was performing using SPSS Version 20 (IBM SPSS Statistics 20, USA). Categorical variables were summarized by proportion and continuous variables were summarized by mean and median with 95%CI and IQR where appropriate. The primary outcomes were overall presumptive MDR-TB cases appropriately accessing molecular diagnostics testing for early diagnosis and treatment in both retreatment TB cases and in presumed TB in PLWH. Pre-specified variables hypothesized to contribute to the primary outcome included knowledge of healthcare workers in implementing international standards for TB diagnostics and capacity of laboratories with molecular diagnostics in providing reliable MDR-TB diagnosis. Variables were compared across the regions and using χ2 or Fisher’ s exact tests, or one-way ANOVA or Kruskal - Wallis tests as appropriate. All statistical tests were two-sided with *p* < 0.05 considered significant. For the qualitative section, data analysis was undertaken by S.G.M. The author read interview texts and familiarized with the content. Using an open code, the author independently identified key issues and the theme from each of the health care workers. Issues were described into excel spreadsheet version 2014 and transferred into SPSS. Results were summarized in number (percentage).

## Results

Twenty-eight districts (51%) were randomly selected from the 55 districts to participate in the study. Only 11 (39% of selected districts) had laboratories with capacity for performing MDR-TB diagnosis with molecular diagnostics and were therefore included. Thus, 28 DTLCs, 28 DACs and 11 Laboratory Managers were eligible. Of those eligible front-line healthcare workers, 64 (96%) participated; including all 11 (100%) of the laboratory managers. A cohort of presumed MDR-TB patients that received services in the previous year either at a TB or HIV clinic was summarized.

### MDR-TB diagnosis clinical practices

**Fig. 2:**
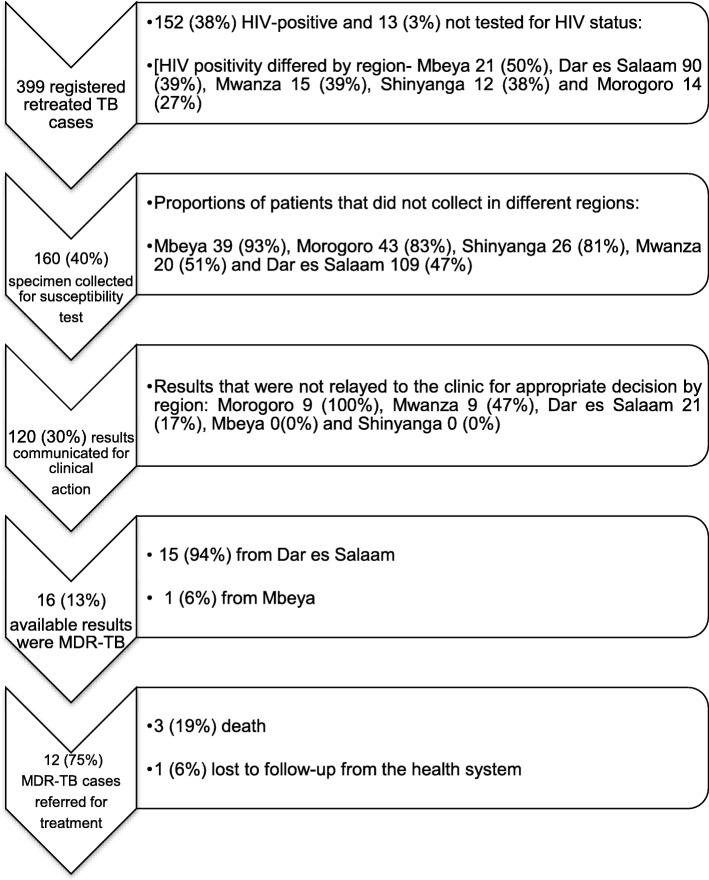
Flow of presumed MDR-TB from diagnosis to treatment in previously treated TB cases in 2015. N.B Shinyanga region contributed only 3 (75%) of the randomized districts while Dar es Salaam combines 3 designated regions (Ilala, Kinondoni and Temeke) with randomized districts within each region

In the retrospective cohort of TB patients, 399 were identified as “retreatment” and at high risk for MDR-TB (Fig. 2). Distribution of the cohort was as follows: the 3 regions of Dar es Salaam included 49 (12%) from Ilala, 97 (24%) from Kinondoni, and 88 (22%) from Temeke; Mbeya contributed 42 patients (11%), Morogoro 52 (13%), Mwanza 39 (10%) and Shinyanga 32 (8%). The mean age was younger in those districts from the Dar es Salaam regions, mean of 38 years (CI 95%CI; 36–39) compared to the other four regions with mean of 40 years (95%CI; 39–42). A higher proportion of males, 294 (74%) were found among the retreatment cases compared to female, 105 (26%). While the mean HIV prevalence was 38%, it reached as high as half of all retreatment cases in Mbeya. Nevertheless, Mbeya also recorded the highest 39 (93%) proportion of retreatment cases that did not have a specimen collected for any type of drug susceptibility testing (molecular diagnostic or otherwise). Among PLWH and presumed MDR-TB, the subset with the highest risk of poor outcome, that proportion that did *not* have a specimen collected for drug susceptibility testing was 20 (95%) in Mbeya, followed by Shinyanga 10 (83%), Morogoro 11 (79%), Mwanza 8 (53%) and Dar es Salaam 42 (47%). In 216 instances (91%) of all patients for whom a specimen was not collected, there was not a specific reason for lack of collection stipulated in the records.

Furthermore, 40 (25%) of submitted specimens did not have results communicated back to the clinic including for 11 (18%) of tests performed in PLWH. In the few with submitted specimens, XpertMTB/RIF was the most common test and used in 86 (72%), while 29 (24%) relied on conventional culture based testing, and none (0%) by the GenotypeMTBDR*plus*. Importantly, only 16 patients (13% of the total 399 with retreatment TB) were ultimately diagnosed with MDR-TB, and of those only 2 (13%) had HIV co-infection compared to 152 (38%) of the total retreatment population with HIV-coinfection. Lastly, only 12 patients successfully started MDR-TB and Fig. 2 illustrates the steps in the process where opportunity for diagnosis, communication of results, and referral to treatment were missed.

**Fig. 3:**
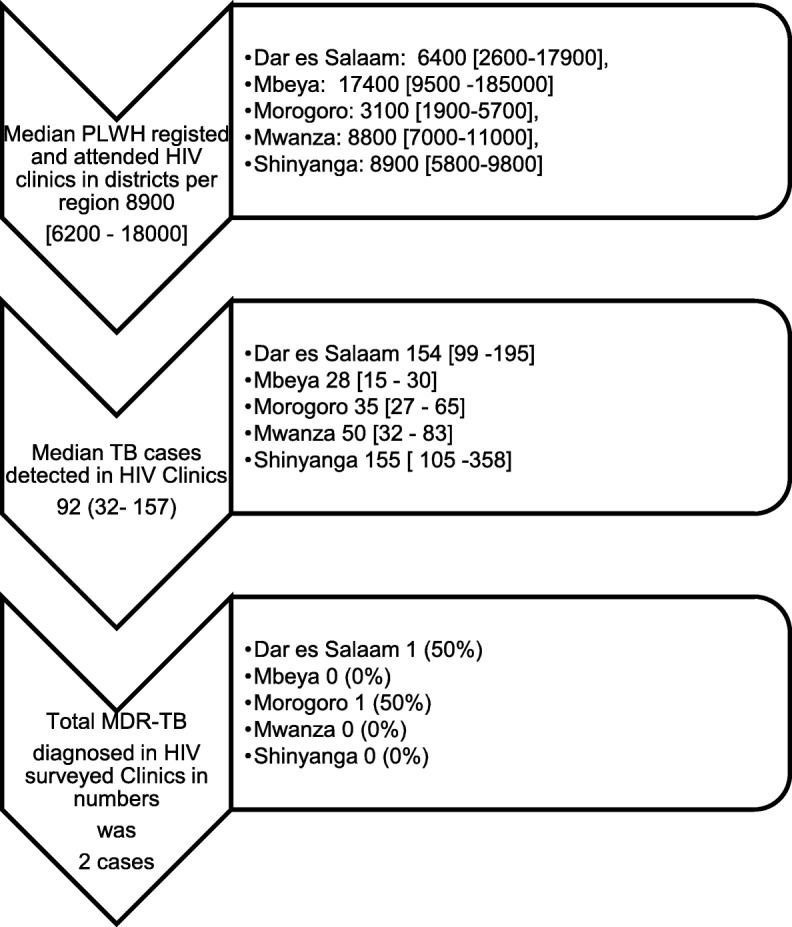
Trend of MDR-TB diagnosis in HIV/AIDS clinics. N.B Dar es Salaam and Shinyanga contributed data of people living with HIV (PLWH) in 11 (92%) and 3 (75%) randomized districts respectively

In further examination of TB services for PLWH, records in the district AIDS Centres from 2015 were specifically reviewed. DACs managed a median of 9 (IQR 5–13) HIV clinics per district. Figure 3 illustrates the steps in the processes of TB and MDR-TB diagnosed from PLWH and attending HIV clinics separated by region, and reflected a vast under diagnosing of MDR-TB relative to the expected burden in the population. We also noted differing DACs’ preferences for use of the diagnostic tests and their level of comfort with initiating TB treatment. When TB was detected in PLWH only 14 (54%) of DACs were able to initiate TB treatment and the remainder preferred to refer patients to TB clinics. DACs were then asked about prior or hypothetical cases of MDR-TB, and 20 (76%) DACs preferred to refer to the district or regional TB coordinators, 3 (12%) would refer to the national MDR-TB hospital, and 3 (12%) admitted that they did not know how they would manage such cases, and this level of preference varied significantly by region.

### Laboratory capacity

Only 11 (39%) districts had laboratories that had the capacity for MDR-TB diagnosis. All were public laboratories and were equipped with the 4 module version of the XpertMTB/RIF machine. None surveyed used the Genotype MDRTB*plus*. Four (36%) and 7 (64%) of XpertMTB/RIF machines were originally provided by the government and donors respectively, although one machine originally provided by the donor was now maintained by the government. The median number of specimens received were higher compared to the number of specimens processed per day throughout the regions. Furthermore, the frequency of laboratory staff performing XpertMTB/RIF that had not received formal training was common in all regions (Table 1). The median months that XpertMTB/RIF machines were not working was higher in Dar es Salaam than in other regions (Table 2).

**Table 1:** Laboratories with molecular diagnostics in surveyed regions

Characteristics	Regions					
	All	Dar es Salaam	Mbeya	Morogoro	Mwanza	Shinyanga
Laboratories with molecular diagnostics for MDR-TB diagnosis per District. No.(%)	11 (39)	5 (42)	2 (50)	1 (25)	1 (25)	2 (50)
Number of samples received for XpertMTB/RIF per day. No/ median (IQR)	12 (11–22)	20 (14–24)	11 (10–12)	12 (−)	10 (−)	21 (11–30)
Number of samples tested in XpertMTB/RIF per day. No/Median (IQR)	12 (12–14)	12 (12–16)	9 (6–12)	8 (−)	10 (−)	18 (12–24)
Number of staff for operating the XpertMTB/RIF per laboratory	3 (2–6)	3 (2–3)	7 (2–11)	2 (−)	5 (−)	9 (7–10)
Number of staff trained for operating the XpertMTB/RIF. No./Median (IQR)	3 (2–4)	3 (2–3)	2 (−)	3 (−)	2 (−)	4 (−)
Estimated months XpertMTB/RIF was not working due to several reasons	2 (1–4)	3 (2–5)	3 (1–4)	2 (−)	1 (−)	2 (1–2)

**Table 2:** Knowledge of molecular diagnostics for MDR-TB comparing DTLCs and DACs

Molecular Technology	Characteristic	Sub Category	All	DTLCs	DACs	p - value
GeneXpertMTB/RIF	Description. No. (%)	Understands	37 (70)	22 (81)	15 (57)	0.11
		Intermediate understanding	14 (26)	5 (19)	9 (35)	
		Does not understand	2 (4)	0 (0)	2 (8)	
	Clinical application. No.(%)	Understands	38 (72)	24 (89)	14 (54)	0.015
		Intermediate understanding	13 (24)	3 (11)	10 (38)	
		Does not understand	2 (4)	0 (0)	2 (8)	
	Result interpretation. No. (%)	Understands	22 (42)	16 (59)	6 (23)	0.014
		Intermediate understanding	15 (28)	7 (26)	8 (31)	
		Does not understand	16 (30)	4 (15)	12 (46)	
GenotypeMTBDR*plus*	Description. No. (%)	Understands	10 (19)	8 (30)	2 (8)	0.12
		Intermediate understanding	12 (23)	5 (19)	7 (27)	
		Does not understand	31 (58)	14 (51)	17 (65)	
	Clinical application. No. (%)	Understands	9 (17)	8 (30)	1 (4)	0.36
		Intermediate understanding	14 (26)	7 (26)	7 (27)	
		Does not understand	30 (57)	12 (44)	18 (69)	
	Result interpretation. No.(%)	Understands	0 (0)	0 (0)	0 (0)	0.15
		Intermediate understanding	5 (9)	1 (4)	4 (15)	
		Does not understand	48 (91)	26 (96)	22 (85)	

*DTLC* District tuberculosis and leprosy coordinator. *DAC* District AIDS coordinator

### Front-line healthcare workers’ familiarity with molecular diagnostics

All front-line healthcare workers’ knowledge of the clinical application and interpretation (Additional file 1) of molecular diagnostics was below expected levels. Only 10 (19%) of DTLCs and DACs were categorized as “able to describe well,” the molecular diagnostic technologies (XpertMTB/RIF and GenotypeMTBDRplus) and 2 (4%) were unable to describe either at all, while all others were partially conversant (*p* = 0.14). Furthermore, only 9 (17%) of both DTLCs and DACs were well acquainted with the clinical application of molecular technologies while 2 (4%) did not score as having competence in either of the technologies. However, levels of proficiency in the clinical application differed by test type as more were noted to be conversant with XpertMTB/RIF, 38 (72%), than GenotypeMTBDR*plus,* 9 (17%), (*p* = 0.003). Combining both DTLC and DAC, 15 (28%) lacked the capacity for interpreting results for either of the molecular diagnostics while only 3 (6%) were able to “interpret well,” and all others were only partially conversant. Yet there was no difference in the distribution of DTLC and DAC in the ability level for interpreting both XpertMTB/RIF and GenotypeMTBDR*plus* (*p* = 0.68). Knowledge distribution of DTLCs and DACs for XpertMTB/RIF and GenotypeMTBDRplus is summarized in Table 2. While, commonly described challenges for front-line healthcare workers to access and utilize MDR-TB diagnostics and support patients to treatment are summarized in Table 3.

**Table 3:** Challenges and bottlenecks identified by front-line healthcare workers

A. DTLCs and DACs identified challenges	Frequencies. n (%)
Lack of specimen referral mechanisms	23 (43)
Unreliable patients address	13 (25)
Shortage of healthcare workers	7 (13)
Patients opt to find traditional medicine and do not return for results	5 (9)
Lack of equipment for diagnostics	3 (6)
Lack of healthcare worker motivation	1 (2)
Rotation to different section regardless of knowledge on TB/HIV	1 (2)
B. Laboratory designated heads	n (%)
Stock out of XpertMTB/RIF cartridges or related reagents	9 (82)
Unreliable maintenance and service if needed	4 (36)
Shortage of staff for operating XpertMTB/RIF	2 (18)
Forced reliance on untrained staff	1 (9)
Few samples for XpertMTB/RIF received per day	1 (9)
Samples received at late hours	1 (9)
Electrical fluctuations	1 (9)

*DTLC* District tuberculosis and leprosy coordinator. *DAC* District AIDS coordinator

## Discussion

**Fig. 4:**
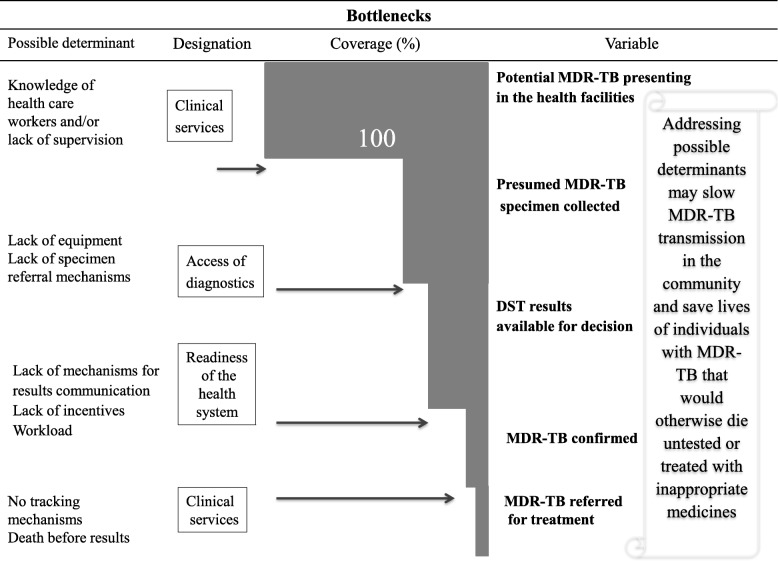
Health system barriers to the implementation of MDR-TB diagnosis in a public health care settings

This study examined three important settings within the healthcare system: TB clinics, HIV clinics and laboratories with MDR-TB diagnostics at the front-line of patient care in Tanzania. In so doing, we identified factors that potentially influenced both a lack of diagnosis and a delay in diagnosis of MDR-TB (Fig. 4). We uncovered widespread underuse of molecular diagnostics for the two specific cohorts at higher risk of MDR-TB and for whom international consensus recommends the use of molecular tests, those with retreatment TB and PLWH. The majority of front-line healthcare workers, as represented by DACs and DTLCs surveyed, demonstrated opportunity for knowledge improvement on the use of molecular diagnostics, and knowledge was more deficient for GenotypeMTBDR*plus* compared to XpertMTB/RIF, and for both technologies in DACs compared to DTLCs. While these knowledge gaps are not dissimilar from recent studies of front-line healthcare workers in other sub-Saharan African countries and elsewhere regarding molecular diagnostics for TB [17], the scale of effect was profound. Only 40% of previously treated TB cases were tested for MDR-TB, lower than the global estimate of 60% [1], and when accounting for the those tested but for whom results were not communicated to the clinic, only 30% of all cases of presumed MDR-TB received the opportunity for the most accurate and effective treatment, which is exceptionally low compared to other settings [18].

Previous proportions of retreatment patients without drug-susceptibility testing in Tanzania were reported as high as 90% from a 9-year audit in 2013 [19], so it can be inferred that availability of molecular diagnostics has slightly improved this testing pattern. Yet we believe the rigor of our standardized data collection methods among randomly selected districts from the 5 highest burdened regions is most reflective of current practice patterns and workforce capacity. As such, the lack of diagnostics utilization was even more concerning when restricted to HIV/AIDS clinics where the vast majority of presumed TB cases were screened using conventional smear microscopy [20], despite well-defined WHO recommendations to use XpertMTB/RIF for improved sensitivity of TB detection and MDR-TB diagnosis [21]. Front-line healthcare workers were further compromised in their ability to deliver the recommended diagnostics secondary to unreliable laboratory services as machines were frequently out of service or cartridges or other consumables out of supply, as well as a lack of standardization and support for the specimen referral mechanisms. Our findings suggest that the current health systems have been slow to implement international standards of TB care surrounding the use of molecular diagnostics and goals to diagnose and treat MDR-TB, which have likely led to ongoing transmission, and without considerable structural change in the healthcare workforce and massive resource influx will never see the benefit of these technological advances [22].

We also noted that even in the cases of successful MDR-TB diagnosis, 20% were lost to follow-up or died prior to treatment initiation. While data for this observation were representative of only one of the regions studied, the median time from results communicated to the clinic to referral for treatment was 5 days (IQR 1–15). This time to referral should not result in early death, and we suspect rather that death was influenced by extensive disease from delay in presentation and delay in ultimate performance of the diagnostic test. We previously reported that extensive lung destruction was associated with a higher early mortality in the first cohort of MDR-TB treated cases [23].

Unfortunately, the majority of DTLCs and DACs were unable to stipulate why molecular diagnostics were not used more frequently, and we interpret this to indicate a generalized lack of ownership over this aspect of the health system. From our local findings, we postulate that initiatives to deliver continuing medical education, such as in the form of modules available on the web and accessed through mobile devices, and linking this training to professional recertification, would vastly increase uptake of new technology. Currently there are no formal training or demonstrations of knowledge required for professional recertification in Tanzania. We also propose transfer of molecular diagnostics ownership and maintenance to public-private partnered management as responsibilities for machine maintenance and consumables provisions are borne by the end-users. When this strategy has been used in other TB endemic settings, diagnostic uptake has been increased and sustained [24].

In addition to proper use of existing molecular diagnostics machines, reflective of the quality of services, we observed, as others have, a considerable need to scale up access, reflective of the quantity of care, as the availability of diagnostics was present in only 39% of the surveyed districts [25]. While the original target was for one XpertMTB/RIF machine in every district in Tanzania, this quantity of machine is still below the number of machines needed to efficiently screen the number of patients with retreatment TB or PLWH and presenting with TB symptoms [26]. Given the current lack of availability and proper use of molecular diagnostics in Tanzania, it remains very difficult to study the benefit of rollout of these technologies to-date. Nevertheless, this type of implementation study may be more important than ever given our observations regarding equipment maintenance and servicing and stock out of consumables, which in at least one case interrupted operation for up to 5 months.

While the retrospective review of programmatic records has inherent limits such as missing information and recall bias, the former was infrequent as exemplified by consistent reporting of demographics and HIV status among retreatment TB cases. We attempted data extraction from records from TB and HIV/AIDS clinics, as well as laboratory records, which also minimized incomplete data. However, some clinical information, such as the number of prior episodes of TB treatment or the number of times a patient had accessed care or diagnostics prior to the time period studied, were not available but may have informed the egregiousness of lack of testing for MDR-TB or the further systems errors in repeating microscopy despite negative or non-diagnostic prior testing. We followed the STROBE guidelines for reporting of results with the intent to provide a policy guide for stakeholders in Tanzania [27]. However, our sampling strategy failed to capture any laboratories or districts utilizing GenotypeMTBDR*plus* which limited lack of interpretation of its use and may explain the lack of knowledge by the DACs and DTLCs with this assay.

## Conclusion

The majority of cases at higher risk for MDR-TB missed the opportunity of early screening, diagnosis and treatment. Identified bottlenecks in this process include a lower level of knowledge of molecular diagnostics for TB among front-line healthcare providers, poor access to molecular diagnostics based on the estimated number of retreatment TB cases and PLWH in the high burden regions surveyed, unreliable equipment maintenance, and inconsistent availability of supplies and consumables. These elements point toward health systems barriers that further remove the patient from the center of TB care decision making, as is advocated in the End TB Strategy [28]. Add to the major epidemic of TB/HIV, the emerging non-communicable disease interaction of TB and diabetes mellitus, and the cycling infectious epidemics such as dengue and cholera, we forecast that health systems in Tanzania responsible for TB care will be ill-equipped to realize the benefit of molecular diagnostics without large-scale changes [29, 30].

## Additional file

Additional file 1: Expected responses for knowledge assessments on molecular diagnostics; XpertMTB/RIF/GenotypeMTB/RIF for MDR-TB diagnosis. (Knowledge on molecular diagnostics). (DOCX 50 kb)

## Abbreviations

DACs: District AIDS (Acquired Immunodeficiency Syndrome) Coordinators,
DTLCs: District TB & Leprosy Coordinators
HIV: Human Immunodeficiency Virus
MDR: Multidrug resistant
PLWH: People Living with HIV
TB: Tuberculosis
WHO: World Health Organization
