# Supplementary material for: Gridlock from diagnosis to treatment of multidrug-resistant tuberculosis in Tanzania: low accessibility of molecular diagnostic services and lack of healthcare worker empowerment in 28 districts of 5 high burden TB regions with mixed methods evaluation
Source: BMC Public Health. 2019 Apr 11;19:395. doi: 10.1186/s12889-019-6720-6 (PMC6458695; doi:10.1186/s12889-019-6720-6)
Supplement: Supplementary file 1 — Expected responses for knowledge assessments on molecular diagnostics; XpertMTB/RIF/GenotypeMTB/RIF for MDR-TB diagnosis. (Knowledge on molecular diagnostics). (DOCX 50 kb) [file 12889_2019_6720_MOESM1_ESM.docx]

**Additional file 1**

**Expected responses for knowledge assessments on molecular diagnostics; XpertMTB/RIF/GenotypeMTB/RIF for MDR-TB diagnosis**

| **XpertMTB/RIF** |  | **GenotypeMTBDRplus** |
| --- | --- | --- |
| **General description of the test** |  | **General description of the test** |
| WHO endorsed rapid molecular diagnostic test |  | WHO endorsed rapid molecular diagnostic test |
| Identify MTB DNA in both smear positive and negative PTB cases |  | Detect MTB in known smear positive PTB |
| Detect resistance of rifampicin |  | Detect resistance of Rifampicin and isoniazid |
|  |  |  |
| **Clinical Application** |  | **Clinical Application** |
| Presumed TB cases suspected to harbor DR-TB regardless of age example all retreatment category |  | Presumed MDR-TB and known smear positive PTB case |
| Presumed TB cases in individuals living with HIV |  | MTB isolates screen/detect MDR-TB |
| Presumed TB and seriously ill regardless of the HIV status |  | Presumed isoniazid mono-resistance and or confirm Rifampicin detected with XpertMTB/RIF |
|  |  |  |
| **Result interpretation and action** |  | **Result interpretation and action** |
| *Error, invalid or no results:*  Test failed Need to repeat |  | ***Invalid test result:***  Test fail to deliver appropriate results. Need to be repeated |
| *MTB not detected:*  Conduct a follow-on diagnostic evaluation especially for symptomatic presumptive cases |  | ***MTB not detected:***  No TB detected however the limitation for sensitivity in smear positive required alternative test like culture or XpertMTB/RIF |
| *MTB detected RIF resistance not detected:*  Start patients a drug sensitive TB regimen |  | ***MTB detected plus RIF and INH resistance not detected:***  This is a diagnosis of a drug sensitive TB and standard short course TB therapy can be initiated (6 months) as per WHO guideline |
| *MTB detected RIF resistance detected:*  Follow the algorithm below to identify the risk factor for MDR-TB |  | ***MTB detected plus RIF and INH resistance detected:^+^***  This is highly suggestive of MDR-TB but needs to be interpreted in the context of risk factors for drug resistance |
|  |  | ***MTB detected plus INH (only) resistance detected:^++^***  This indicates possible mono resistance to INH |

| + If the patient has been previously treated for TB or has failed first line treatment, then risk of MDR-TB is high. In such a patient, a positive rifampin (RIF) resistance result is sufficient to initiate second-line therapy for MDR-TB. At the same time, a sample should be sent for liquid culture and complete drug susceptibility testing (DST). When the DST results come back in about 2 – 3 weeks, the MDR-TB regimen can be modified/customized, based on DST profile. |
| --- |
| ++ While INH mono-resistance is not MDR-TB, such patients may have a higher risk of poor outcomes and amplification of drug resistance. Thus, a liquid culture *plus* full DST should be ordered to customize treatment. |
